# Techno-Economic and Statistical Assessment of Agricultural Flours for Bacterial Cellulose Production by *Komagataeibacter xylinus*

**DOI:** 10.3390/polym18060721

**Published:** 2026-03-16

**Authors:** Dheanda Absharina, Csilla Veres, Sándor Kocsubé, Csaba Vágvölgyi

**Affiliations:** Department of Biotechnology and Microbiology, Faculty of Science and Informatics, University of Szeged, 6726 Szeged, Hungary; dheanda.absh@gmail.com (D.A.); gmri.csilla@gmail.com (C.V.); shigsanyi@gmail.com (S.K.)

**Keywords:** bacterial cellulose, *Komagataeibacter xylinus*, nitrogen substitution, low-cost media, techno-economic analysis, sustainable fermentation

## Abstract

Nitrogen supplements such as yeast extract and peptone/tryptone are the main cost drivers in bacterial cellulose (BC) fermentation. This study evaluated fourteen cereal, pseudo-cereal and legume flours as media substitutes for *Komagataeibacter xylinus* DSMZ 2325 using two strategies: (i) constant total nitrogen (CTN; 0.6 g·L^−1^) and (ii) constant nitrogen-source mass (CNSM; 5.0 g·L^−1^). BC yield (dry g·L^−1^) was determined under static cultivation and analyzed by ANOVA, correlation statistics and techno-economic assessment. Flour type and substitution level significantly influenced BC production (*p* < 0.05). CTN substitution enhanced production, with the highest peak yields obtained for W-BC, C-BC, M-BC and SP-BC (6.68–8.97 g·L^−1^). CNSM substitution limited production, with O-BC and T-BC performing best (4.24–5.14 g·L^−1^). Techno-economic analysis further showed that the CTN regime substantially improved cost efficiency and reduced BC unit production cost, with the maximum reduction observed for TR-BC at 75% substitution (from 0.27 to 0.08 €/g; 70.37%) relative to the corresponding CTN HS control. Under the CNSM regime, the maximum reduction was observed for BY-BC at 50% substitution (from 0.25 to 0.07 €/g; 72.00%) relative to the corresponding CNSM HS control. These findings demonstrate that graded nitrogen substitution is an effective strategy for economically sustainable and scalable BC production.

## 1. Introduction

In kombucha beverage production, the cellulose pellicle is obtained as a low-value coproduct with limited direct economic return. Nevertheless, this kombucha-derived cellulose has attracted increasing interest because, as a microbially synthesized material, it often exhibits superior structural and functional properties compared with plant-derived cellulose. As a result, kombucha-BC has been investigated for biopolymer-based films to enhance mechanical and barrier performance and has also been explored as a renewable material for sustainable packaging applications. Reflecting this growing interest, recent studies by Treviño-Garza et al. (2020), Nguyen et al. (2021), and Hernández et al. (2024) examined biopolymers produced by kombucha-associated microbial consortia and highlighted their potential for commercial use [1,2,3].

More broadly, bacterial cellulose (BC), first reported in 1886 in the context of vinegar fermentation, has sustained research because of its distinctive physicochemical properties and expanding application potential. Produced extracellularly by acetic acid bacteria, particularly *Komagataeibacter xylinus*, BC consists of highly crystalline β-1,4-glucan microfibrils in a unique three-dimensional nanostructure, leading to high tensile strength, water-holding capacity, and biocompatibility, all qualities which support BC’s applications in food structuring, wound dressings, biomedical scaffolds, and flexible electronics [4]. Therefore, BC is no longer viewed solely as a laboratory-scale material but is already manufactured industrially for selected high-value applications.

Despite these advantages, wider industrial implementation of BC remains constrained by production cost and process efficiency. A major contributor to this limitation is the composition of the cultivation medium, particularly under static fermentation, which is still widely preferred for obtaining high-quality pellicles. Medium formulation directly affects carbon and nitrogen availability, redox conditions, oxygen transfer, cellulose yield, and fibrillar network development while also representing a substantial proportion of upstream production cost [4,5]. In static systems, additional dependence on oxygen transfer at the air–liquid interface further limits volumetric productivity, reinforcing the need for economically efficient medium design and process optimization.

Accordingly, recent BC research has increasingly focused on three complementary strategies: development of engineered strains, replacement of refined substrates with low-cost or waste-derived feedstocks, and improvement of bioprocess performance through fermentation and downstream optimization [5]. Although strain engineering may improve productivity, it often requires longer development timelines and introduces regulatory complexity. In contrast, substrate substitution and process optimization are more immediately applicable for reducing production cost under defined operating conditions.

Within this cost structure, refined nitrogen supplements such as yeast extract and peptone/tryptone represent a significant medium expense because they are commonly required to sustain *Komagataeibacter* growth and cellulose biosynthesis [5,6]. In parallel with efforts to reduce nitrogen-related costs, growing attention has also been directed toward replacing refined carbon sources with lower-cost alternatives. For example, Alidu and Alexander (2026) reported that mixed fruit-and-vegetable food waste could serve as an effective low-cost carbon source for kombucha fermentation, producing BC yields higher than refined-sugar controls while maintaining characteristic functional groups and favorable crystallinity [7]. Similarly, sweet black tea-based media supplemented with ethanol and inorganic nitrogen sources such as ammonium sulfate have been optimized to achieve high BC titers within shortened cultivation times under static conditions [8].

To further reduce medium cost, numerous studies have explored minimally processed agro-derived substrates and agro-industrial side streams, including fruit and vegetable by-products, molasse hydrolysates and lignocellulosic residues [9,10,11]. Cereal fractions have also been considered for a similar role [12]. Multiple studies proposed spent tea as alternative nutrient sources for BC production [13,14,15]. These approaches are consistent with circular bioeconomy principles because they valorize low-value residues while reducing reliance on food-grade substrates [4,5,16]. From a substrate perspective, agro-industrial materials offer a clear opportunity to lower medium cost while providing carbon and other nutrients required for microbial growth and cellulose synthesis [14]. However, their application is not without limitations. Their heterogeneous and often undefined composition can compromise process reproducibility, alter bacterial growth behavior, and lead to variation in cellulose yield and product quality [12]. In addition, inhibitory-compound-derived fermentable substrate may suppress fermentation efficiency.

To improve substrate suitability, certain waste-derived feedstocks require pretreatment, including acid or enzymatic hydrolysis, to depolymerize complex polysaccharides and increase fermentable sugar availability. Although such pretreatments may improve BC production, they also introduce additional operating costs, process complexity, and, in some cases, further purification requirements. This balance between opportunity and process burden is illustrated by El-Gendi et al. [17], who reported that enzymatically hydrolyzed prickly pear peels supported a BC yield of 6.01 g·L^−1^ under optimized conditions, while BC membranes functionalized with fruit by-products such as pomegranate peel extract exhibited antimicrobial activity and extended strawberry shelf life, indicating potential for sustainable packaging applications. These findings demonstrate that waste-derived substrates can support both cost reduction and product innovation but also underpin that the cost related culture media utilization must be interpreted together with pretreatment and downstream requirements.

Among potential low-cost nutrient sources, cereal, pseudo-cereal, and legume flour represent particularly relevant candidates because they are abundant within agricultural processing chains and contain substantial amounts of protein, starch, minerals, and micronutrients that may support microbial metabolism [18]. Importantly, the refined nitrogen supplements targeted for replacement are themselves derived from processed agricultural resources. Accordingly, the present study does not advocate diversion of primary food resources but rather evaluates bulk or off-specification fractions within established supply chains. From an industrial biotechnology perspective, such materials are relevant because they are comparatively stable, widely available, and less compositionally extreme than many heterogeneous waste streams, thereby offering a potentially more efficient basis for medium reformulation.

Systematic evaluation of nitrogen substitution strategy, controlled comparison of replacement frameworks and cost-normalized assessment of performance remain comparatively underdeveloped in BC research. This limitation is significant because increased production alone does not establish economic viability. Industrial relevance depends not only on yield but also on unit production cost, raw material price stability, supply chain reliability, and process robustness under defined conditions [16,19]. Nevertheless, techno-economic analysis (TEA), despite being widely recognized as an essential decision-support tool in industrial biotechnology, remains underrepresented in BC medium development studies and is often applied only after completion of experimental work rather than being integrated prospectively into experimental design. Earlier TEA integration is therefore necessary to distinguish actual cost reduction from merely apparent gains in productivity.

A previous study [20] demonstrated the technical feasibility of partially substituting refined nitrogen sources with thirteen agro-derived flour variants and confirmed the structural compatibility of the resulting BC through physicochemical characterization of selected high-yielding formulations. However, the quality-related findings reported previously apply only to the selected high-yielding formulations that were directly compared with the HS control and should not be generalized to all flour-containing media. Therefore, the feasibility of alternative media should be interpreted based on both productivity and BC quality relative to the corresponding control rather than yield improvement alone. Related work has also shown that cereal-derived nitrogen substrates can influence BC crystallinity, fibril diameter, and thermal stability while maintaining cellulose productivity [21]. The question of nitrogen replacement strategies, however, remains insufficiently explored. Likewise, the economic consequences of such substitution strategies have not yet been evaluated.

To address both the methodological and techno-economic gaps in current BC medium research, two nitrogen replacement strategies were defined and compared: (i) maintenance of constant total nitrogen (CTN) across formulations and (ii) maintenance of constant nitrogen-source mass (CNSM) irrespective of nitrogen equivalence. Each strategy was evaluated at substitution levels of 25%, 50%, and 75% relative to refined nitrogen controls. This design enables direct discrimination between nitrogen-equivalence effects and composition-driven effects of flour-derived substrates on BC synthesis. To strengthen industrial relevance, experimental performance was further integrated with statistical modeling and prospective techno-economic normalization to unit production cost (€/g). In addition, a simulation-based baseline scenario covering seed preparation, static fermentation, purification, and drying was used to identify cost-sensitive process elements and to compare substitution scenarios within defined system boundaries. This approach provides a more rigorous basis for identifying nitrogen substitution conditions that are both technically feasible and economically meaningful.

## 2. Materials and Methods

### 2.1. Strain Maintenance and Inoculum Preparation

*Komagataeibacter xylinus* DSMZ 2325 (German Collection of Microorganisms and Cell Cultures, DSMZ, Braunschweig, Germany), available from the Szeged Microbiology Collection SZMC 28880, was used as the BC-producing strain due to its high cellulose production and adaptability to both static and agitated culture conditions [22]. The strain was cultivated on modified Hestrin–Schramm (HS) agar and stored at 4 °C. For seed culture preparation, biomass from a 7-day plate culture was transferred into 50 mL of HS broth and incubated at 28 °C and 120 rpm for 48 h. Actively growing cultures (OD_600_ = 0.7–1.0) were used as 10% (*v*/*v*) inoculum in all fermentation experiments to ensure consistent initial cell density across treatments.

### 2.2. Media Formulation and Flour Variant Preparation

The control medium was a modified Hestrin–Schramm (HS) formulation containing sucrose (80.0 g·L^−1^), yeast extract (2.5 g·L^−1^), and tryptone (2.5 g·L^−1^). In the experimental media, yeast extract and tryptone were partially replaced with selected flour variants. Unless stated otherwise, no additional pH adjustment was applied after medium preparation. To systematically evaluate nitrogen substitution, two complementary experimental strategies were implemented based on recent findings revealing that certain agro-derived flour variants can function as alternative nitrogen sources in HS-based media [20]. The first strategy maintained a constant total nitrogen concentration (CTN) of 0.6 g·L^−1^ across all formulations. Under this regime, yeast extract, tryptone and flour quantities were recalculated so that the particular flour would substitute 25%, 50%, or 75% of the media while also maintaining a 0.6 g·L^−1^ total nitrogen content. The second strategy maintained a constant nitrogen-source mass (CNSM) by applying a fixed media weight concentration of 5.0 g·L^−1^. In this case, substitution levels of 25%, 50%, and 75% were defined relative to the total mass of yeast extract and tryptone replaced. Unlike the CTN regime, this approach maintained a constant flour mass rather than a constant nitrogen input, thereby allowing nitrogen availability to vary as a function of flour composition. The nitrogen content of each flour variant was determined using the relevant literature, which was later supplemented by experimental results obtained from the Kjeldahl method for the purpose of comparison (Supplementary Table S5) [20].

Stepwise substitution levels (25%, 50%, and 75%) were defined to enable controlled evaluation of BC production responses under progressive replacement of refined nitrogen sources while retaining residual yeast extract and tryptone to preserve formulation stability. These substitution levels represent low, intermediate, and high partial replacement regimes. Complete (100%) substitution was previously assessed [20]; therefore, the present study focuses on defining a reproducible and industrially relevant partial substitution range. A detailed overview of flour quantities applied under both the CTN (0.6 g·L^−1^ fixed nitrogen content) and CNSM (5.0 g·L^−1^ fixed media weight) regimes is provided in Table 1.

### 2.3. Fermentation Procedure

Static fermentation was performed as previously described in Absharina et al. [20] using the media formulations detailed in Section 2.2. Sterile glass jars (50 mL capacity) containing 45 mL of medium were inoculated with 5 mL of actively growing seed culture (10% *v*/*v*; OD_600_ = 0.7–1.0), resulting in a total working volume of 50 mL. Media were sterilized at 121 °C for 20 min prior to inoculation. Cultures were incubated at 28 °C for 7 days under static conditions to promote pellicle formation at the air–liquid interface. Static cultivation was selected to replicate the air–liquid-interface-driven cellulose biosynthesis characteristic of nata-type production systems. This configuration enables direct evaluation of medium composition effects without hydrodynamic shear interference and remains a reference mode in industrial BC production and techno-economic modeling studies [23,24].

Fermentation broth pH was measured at initial inoculation (pH_0_) and after 7 days (pH_7_) of cultivation in triplicate using pH indicator strips (Macherey-Nagel GmbH & Co. KG, Düren, Germany; range 4.0–7.0). All experiments were conducted in triplicate.

### 2.4. Purification and Dry Weight Determination

BC pellicles formed at the air–liquid interface were harvested and purified in 0.5 M NaOH (100 °C, 30 min) to remove bacterial cells and residual medium components. Purified pellicles were washed repeatedly with deionized water until neutral pH was achieved and then oven-dried at 120 °C to constant mass and weighed using an analytical balance. BC yields, expressed in g·L^−1^, were calculated by dividing the dry pellicle mass (g) with the initial culture volume (working volume of 50 mL per vessel). Comparable NaOH-based purification, washing-to-neutrality, and dry-weight yield quantification procedures have been widely applied in recent BC studies [20,25,26].

### 2.5. Techno-Economic Analysis

Techno-economic analysis is required to evaluate industrial applicability through process simulation under optimized conditions [16,18]. A comparative techno-economic analysis (TEA) was implemented to quantify the cost implications of graded nitrogen substitution under laboratory-scale static cultivation. The economic system boundary was defined at the culture-medium level and included raw material costs associated with carbon and nitrogen sources. Capital expenditures (CapEx), labor, utilities, depreciation, and downstream processing costs were excluded, as these parameters were identical across experimental conditions and the objective of the present study was comparative evaluation of nitrogen source substitution efficiency rather than full industrial plant modeling [24,27].

Raw material prices (€/g) for sucrose, yeast extract, tryptone, and flour variants were obtained from regional bulk supplier quotations representative of agro-industrial procurement conditions in Central Europe. All prices were normalized to cost per liter of medium (€/L). This approach aligns with contemporary TEA methodology, in which experimentally derived performance data are normalized to enable cost-driven process comparison [16,24]. By isolating medium-dependent operating costs, the analysis enables direct identification of economically favorable nitrogen substitution regimes. The following paragraphs present the calculations used for TEA.

The total medium cost per liter (c_medium_, €/L) for a particular substitution regime was calculated by adding together the cost of all subcomponents in a particular substitution regime:

$$c_{medium}=c_{sucrose}+c_{yeast}+c_{tryptone}+c_{flour}$$

where the cost of subcomponent “i” (c_i_, €/L) was calculated by multiplying its bulk price (p_i_, €/g) with its normalized weight (m_i_ (g·L^−1^)):


$$c_{i}=p_{i}\times m_{i}$$

The unit production cost of BC (C_BC,j,_ €/g) was then calculated for every “j” substitution regime using the total medium cost and the experimentally determined dry pellicle yield (y_BC_, g·L^−1^) measured under the given substitution regime:


$$C_{BC,j}=\frac{c_{medium}}{y_{BC}}$$

The inverse of the unit production cost was defined in the analysis as “cost efficiency” (E_cost_, g/€), because its value gets higher as the pellicle weight produced from expending one price unit (1 €) increases:


$$E_{cost}=\frac{y_{\mathrm{BC}}}{c_{medium}}$$

Comparison with the control was performed using unit cost reduction (%), defined as the relative decrease in BC unit production cost for a given flour substitution case compared with the corresponding HS control within the same substitution regime (CTN or CNSM):


$$cost\;reduction\;(\%)=\frac{\left(C_{{BC}_{control}}-C_{BC,j}\right)}{C_{{BC}_{control}}}$$

Thus, a positive unit cost reduction value indicates that the flour-based formulation achieved a lower BC unit production cost than the regime-matched HS control.

### 2.6. Statistical Analysis

All experiments were performed in triplicate, and results are reported as mean values. Statistical analysis and plots were generated in GraphPad Prism v10.2.3 (GraphPad Software, Boston, MA, USA). Correlation heatmaps were generated using RStudio v2022.02.0 (Posit Software, PBC, Boston, MA, USA). Statistical significance was defined as *p* < 0.05.

### 2.7. Schematic Diagram of Experiment

**Figure 1:**
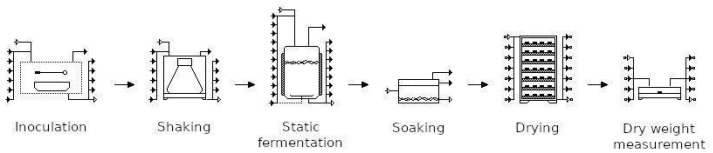
Schematic workflow of static bacterial cellulose production and downstream processing under fixed nitrogen content (CTN) and fixed media weight (CNSM) substitution strategies.

During the experiment, BC was produced using sucrose as the primary carbon source and cereal, pseudo-cereal or legume flours as alternative nitrogen sources. The overall experimental workflow is illustrated in Figure 1 and comprises six sequential unit operations, which include upstream cultivation and downstream processing for yield determination. The upstream stage began with seed culture preparation. Inoculum was cultivated under agitation in sucrose-based medium supplemented with the respective flour variant to stimulate cellulose synthesis. Subsequently, 10% (*v*/*v*) of the seed culture was transferred into static fermentation vessels containing the corresponding fermentation media. Static fermentation was conducted to enable cellulose pellicle formation at the air–liquid interface, where oxygen-dependent oxidative metabolism drives biofilm production. The downstream processing involved alkaline purification of the harvested pellicles. The cellulose sheets were thoroughly washed with distilled water until neutral pH was reached and then dried to constant weight for gravimetric yield determination. The defined system of upstream cultivation and downstream processing is depicted in Figure 1, which was constructed using SuperPro Designer v9.5 (Intelligen, Inc., Scotch Plains, NJ, USA).

## 3. Results and Discussion

### 3.1. BC Production Under the CTN (0.6 g·L^−1^ Fixed Nitrogen Content) and CNSM Regimes (5 g·L^−1^ Fixed Media Weight)

Partial replacement of yeast extract/tryptone with cereal, pseudo-cereal and legume flours resulted in distinct, flour-specific differences in BC yields by *Komagataeibacter xylinus* under both substitution concepts (CTN and CNSM). For the purpose of effective comparison, the overall response patterns of flours under the different substitution regimes were categorized either as “strong”, “moderate” or “weak” depending on the observed yield changes compared to the control (Table 2).

If the yield increment achieved by the flour replacement compared to the control was >100% under at least one substitution level (25%, 50% or 75%) of the particular substitution regime, the flour’s general response under the regime was defined as “strong”. If the yield increment was >25% and ≤100% in the case of at least two substitution levels, the response was defined as “moderate”. If the highest yield increment of a particular substitution regime was ≤25%, the response was considered “weak”.

These categories align with previous studies on BC production, in which yield increments around 100% were classified as “significant” and “remarkable” [16,28,29]. In another study, the potential of a substitution material with a 30% yield increment was considered “substantial” [21], while an increment of 10% or 20% was defined only as a “slight” improvement [13].

#### 3.1.1. CTN Regime: Fixed Nitrogen Content Replacement

Under the CTN regime, in which total nitrogen was fixed at 0.6 g·L^−1^, BC production was generally highest at intermediate substitution levels. Several 50% substitutions resulted in increased BC yields relative to the HS control, with the highest yields obtained for W-BC (8.97 g·L^−1^), C-BC (7.92 g·L^−1^), M-BC (7.51 g·L^−1^), and SP-BC (6.68 g·L^−1^). Across most flours, increasing substitutes from 50% to 75% rarely provided additional gains and frequently led to reduced BC yields. This decline in increment is consistent with solid-associated mass-transfer limitations and reduced nutrient accessibility characteristic of minimally processed plant components in static BC systems, where insoluble starch and fiber fractions increase medium heterogeneity and restrict oxygen diffusion at the air–liquid interface [22,30,31]. Excessive solid loading can impair effective oxygen transfer and reduce cellulose synthase activity. Importantly, statistical comparison (*p* < 0.05) confirmed that multiple CTN formulations achieved yields equal to or significantly higher than the HS control. These findings indicate that controlled nitrogen normalization preserves metabolic balance.

#### 3.1.2. CNSM Regime: Fixed Media Weight Replacement

In contrast, under the CNSM regime (5 g·L^−1^ fixed media weight), BC production was more constrained. The highest yield was observed for O-BC at 50% substitution (5.14 g·L^−1^), followed by T-BC at 25% substitution (4.24 g·L^−1^). At 75% substitution, yields declined across most flours. Unlike CTN, CNSM does not control nitrogen equivalence. As substitution increases, assimilable nitrogen becomes progressively limited, altering the C/N ratio. Both excessively low and excessively high C/N ratios can suppress cellulose biosynthesis by redirecting metabolic flux toward biomass formation or by limiting precursor availability for UDP-glucose synthesis [6]. Additionally, higher flour loading increases medium viscosity and reduces oxygen diffusion, compounding productivity constraints [22].

Overall, protein-rich flours were more resilient than low-protein flours, highlighting the importance of maintaining an appropriate carbon-to-nitrogen (C/N) balance for efficient BC synthesis [20]. The decline in production at high substitution levels under both regimes is consistent with the limited capacity of *Komagataeibacter* to depolymerize untreated starch-rich components, as well as with viscosity-driven oxygen transfer constraints at the air–liquid interface [22,30]. Collectively, these results indicate that 25–50% substitution represents a practical operating range for minimally processed flours. Further improvements may be achievable through mild pretreatment or enzymatic hydrolysis to enhance nutrient accessibility, as suggested in previous substrate-focused and hydrolysate-based studies [28,30,31].

During the experiment, initial pH values (5.0–6.5) reflected flour-dependent buffering capacity; however, after 7 days, all treatments converged to 3.8–5.2 without extreme acidification (<3.5). This decline is consistent with previously observed oxidative glucose metabolism and gluconic/acetic acid formation by *Komagataeibacter* spp. [29,32,33]. Acid accumulation is typically most pronounced within the first 3–4 days [29,34] and, when excessive, can suppress carbon uptake, cellular growth, and cellulose synthase activity [35,36]. Importantly, final pH values remained within the physiologically favorable range (4.0–6.0) reported for optimal BC production [34,37], indicating that inhibitory acid stress was not reached.

### 3.2. Effect of Flour Substitution Level on BC Yield

**Figure 2:**
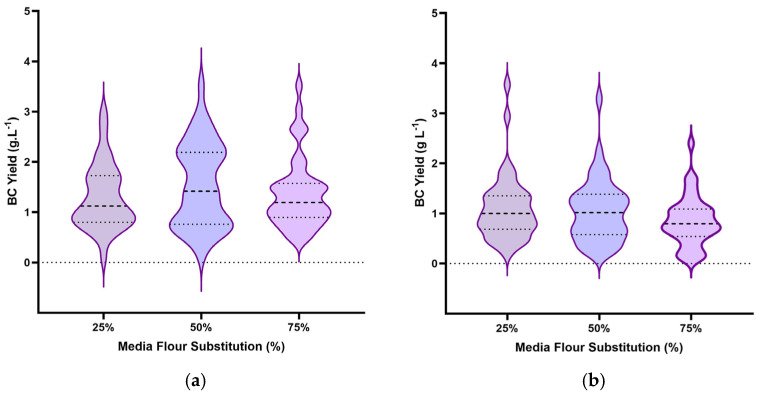
Violin plots illustrating BC yield distributions across 25%, 50%, and 75% flour substitution levels under (**a**) the CTN regime (0.6 g·L^−1^ fixed nitrogen content; **left**) and (**b**) the CNSM regime (5 g fixed media weight; **right**).

The violin plots (Figure 2) depict BC yield distributions across substitution levels, highlighting regime-dependent differences not only in central tendency but also in distributional spread and the presence of high-yield outliers. Under the CTN regime (Figure 2a), BC yield distributions remained centered at or above the HS baseline across all substitution levels. The 50% substitution level exhibited the widest distribution and highest upper-tail outliers, indicating that multiple flour types achieved yields substantially exceeding the control. In contrast, the 75% substitution level displays a narrower distribution and reduced upper quartile, indicating mass-transfer or diffusion limitation due to solid components. Although total nitrogen remains constant, a higher flour proportion contributes to the accumulation of insoluble components, which impede oxygen diffusion and limit nutrient accessibility in static cultivation [12,28]. From a performance perspective, the median yield remained higher than the HS control, confirming that nitrogen normalization sustains overall process stability despite increasing flour proportions.

In contrast, the CNSM regime (Figure 2b) showed a narrower response range at 25–50% substitution and a downward shift in BC yield at 75% substitution. Distribution narrowing at higher substitution indicates a systemic constraint rather than flour-specific variability. As total nitrogen decreases proportionally with refined supplement reduction, the system transitions toward nitrogen limitation while simultaneously experiencing an increased solids concentration. In static BC fermentation, increased medium viscosity and reduced nitrogen accessibility can collectively suppress production. Unlike the CTN approach, the CNSM design does not separate nitrogen availability from solids concentration, which likely explains the more pronounced decline in yield.

The substitution regime comparison shows that while the nitrogen-equivalent CTN substitution can sustain high yield across all substitution levels, the fixed-media-weight CNSM substitution creates limitations and constraints as the substitution rate increases.

### 3.3. Correlation Patterns Between Substitution Level and BC Production

**Figure 3:**
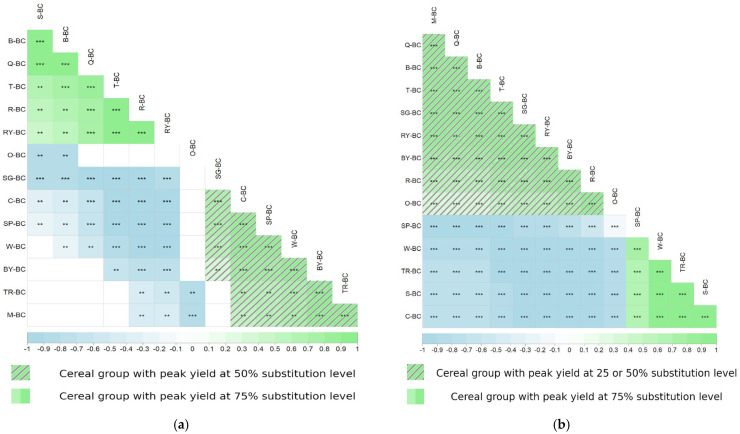
Spearman’s correlation heatmap illustrating the relationship between flour substitution level and BC yield under (**a**) the CTN (0.6 g·L^−1^ fixed nitrogen content) and (**b**) the CNSM (5 g·L^−1^ fixed media weight) regimes. Positive correlations are indicated in green, and negative correlations are indicated in blue. The intensity of the color reflects the strength of the correlation coefficient (r). Asterisks denote statistical significance (* *p* < 0.05; ** *p* < 0.01; *** *p* < 0.001). Flour codes: S (soy), C (corn), W (wheat), R (rice), O (oat), B (bulgur), SP (spelt), Q (quinoa), T (teff), M (millet), TR (triticale), RY (rye), SG (sorghum), BY (barley).

The Spearman rank correlation heatmap (Figure 3) clarifies flour-specific responses across substitution levels. Under the CTN regime (Figure 3a), several flours, including W-BC, SP-BC, TR-BC, BY-BC, and SG-BC, showed strong positive correlations around moderate substitution levels (25–50%), indicating that partial replacement at these levels supports enhanced cellulose production. In contrast, under CNSM (Figure 3b), most flours exhibit neutral-to-negative correlations at high substitution (75%), reflecting a consistent decline in yield as substitution increases. Similarly to the violin plot, the heatmap reveals how substitution with a fixed media weight progressively limits production. However, the comparatively high-nitrogen SP-BC and TR-BC retained positive associations even at higher substitution levels, implying that higher intrinsic nutrient accessibility can partly mitigate these constraints.

The maximum BC yield observed under the CTN regime (8.97 g·L^−1^ for W-BC at 50% substitution) falls within the upper range reported for agro-derived substrates under static cultivation. Previous studies using media based on similar agricultural products recorded comparable results: 1.00 g·L^−1^ for soy flour hydrolyzate [38], 0.70 g·L^−1^ for corn starch hydrolyzate and 2.80 g·L^−1^ for rice starch hydrolyzate, respectively [39].

Alternative media such as rice bran [12], fruit and vegetable residues [9,17], tea by-products [13,15], and molasses-derived substrates [10,23] have supported BC production in the comparable low-to-moderate yield range under static cultivation, although the reported values vary substantially with strain, substrate composition, and pretreatment strategy [4,28,39]. The present work thus demonstrates that nitrogen-equivalent substitution using minimally processed flours can achieve comparatively high yields without enzymatic hydrolysis, chemical preconditioning or pre-selection. Based on the experimental results, the flours studied here have the potential to effectively substitute for other agro-product hydrolyzates or specially treated agricultural or food industry waste [4,11,27]. The direct comparison of CTN and CNSM strategies demonstrates how metabolic nitrogen balance and flour mass concentration can both influence BC production in static systems.

### 3.4. Techno-Economic Analysis of Flour-Based Nitrogen Substitution

Techno-economic analysis (TEA) was conducted following biological validation of medium performance. Only formulations that demonstrated improved BC yields relative to the corresponding HS control were considered for economic interpretation. Unit production cost was expressed as total medium cost per produced BC pellicle weight (€/g). Cost efficiency was defined as produced BC pellicle weight per total medium cost (g/€), i.e., the inverse of unit production cost. To evaluate actual cost reduction, unit cost reduction (%) was also calculated as the percentage decrease in BC unit production cost relative to the corresponding HS control within the same substitution regime (CTN or CNSM).

These metrics were calculated for all flours under both CTN and CNSM conditions using cereal price data from the Hungarian Central Statistical Office [40] and the European Commission’s Agri-Food Data Portal [41], quinoa import price data from the Dutch Center for the Promotion of Imports [42], and reagent prices obtained from Sigma-Aldrich [43,44,45] (Supplementary Tables S3 and S4).

#### 3.4.1. Cost Performance Under Nitrogen-Limited Conditions (0.6 g N)

**Figure 4:**
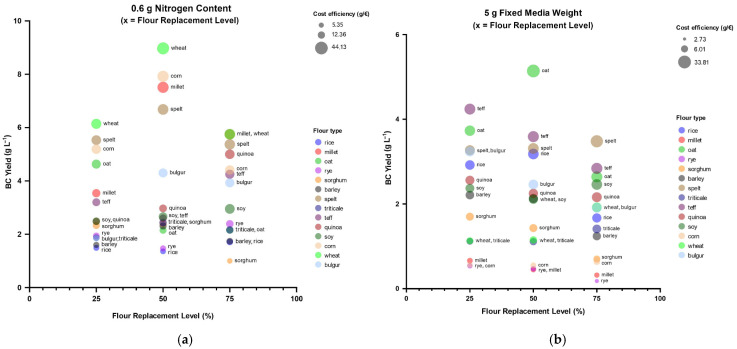
Techno-economic evaluation of BC productivity under CTN and CNSM substitution regimes with substitution levels (25%, 50%, and 75%) for all fourteen flour types. (**a**) Fixed nitrogen content (CTN) of 0.6 g·L^−1^; (**b**) 5 g·L^−1^ fixed media weight (CNSM). Bubble size represents cost efficiency (g/€); unit production cost (€/g) is reported in Supplementary Table S4.

Under the CTN substitution regime (Figure 4a), several widely available flours demonstrated high productivity. W-BC, C-BC, M-BC and SP-BC constituted the best-performing group from the perspective of TEA. The substitution response of these cereals was either strong or moderate, and their cost efficiency ranged from 16.39–44.13 g/€, reflecting high peak yields (6.68–8.97 g·L^−1^) combined with low bulk prices (0.07–0.12 €/g). S-BC, TR-BC and BY-BC (peak yields 2.31–2.95 g·L^−1^) formed the second-most-productive group, demonstrating strong or moderate responses with cost efficiency values of 7.37–26.35 g/€ despite higher bulk prices (0.06–0.17 €/g). Similar values of 7.09–24.93 g/€ were also recorded for T-BC and Q-BC, even though these two were the most expensive flours studied in the experiment (0.57 €/g, 1.01 €/g respectively). The reason for this surprising result is that the cost increase caused by the high flour prices was limited enough to be offset by comparatively high peak yields (4.25–5.00 g·L^−1^).

O-BC, B-BC and SG-BC constituted the third, moderately performing group (peak yields 2.40–4.63 g·L^−1^) with a cost efficiency range of 5.35–21.45 g/€. Although most in this group were classified as weak responders, they were still able to improve cost efficiency compared to the control because of acceptable yields and low bulk prices (0.06–0.11 €/g).

The fourth, weakly performing group (peak yields 1.71–2.39 g·L^−1^) was made up of RY-BC and R-BC, two cereals with weak substitution responses and a low cost efficiency range 6.79–12.79 g/€. In addition to having low yields, RY-BC and R-BC underperformed the control at all substitution levels under the CTN regime.

For the first two groups presented above, unit production cost decreased substantially compared with the HS control, up to 69.50% observed for TR-BC based at 75% substitution (Table S4A; for the formula, see: Section 2.5), while the third group reached a maximum decrease of 58.87% for SG-BC at 75% substitution. These results confirm that moderate nitrogen replacement under CTN conditions can translate into meaningful medium-cost savings without compromising BC yield.

#### 3.4.2. Cost Performance in the Fixed-Media-Weight (5 g) Regime

Under the CNSM regime (Figure 4b), apparent medium-cost savings achieved by replacing yeast extract and tryptone were frequently offset by reduced BC yields under fixed-media-weight conditions. An exception to this tendency was the best-performing flour group, consisting of SP-BC, O-BC and R-BC, with a cost efficiency range of 14.18–33.81 g/€. Even though the substitution responses of these cereals were classified as moderate or weak, their high peak yields (3.18–5.14 g·L^−1^) and low bulk flour prices (0.07–0.19 €/g) proved effective to counterbalance their weaknesses. The expensive, moderate responder T-BC (peak yield 4.24 g·L^−1^) also reached a comparable cost efficiency of 22.42–23.37 g/€. Just as in the case of the CTN regime, the flour’s production-boosting qualities were enough to offset the cost increase caused by its high price (0.57 €/g). S-BC, TR-BC and B-BC (peak yields 1.41–3.24 g·L^−1^), cereals classified either as moderate or weak, constituted the second-best-performing group, with a cost efficiency range of 5.97–20.92 g/€ and low bulk prices (0.08–0.17 €/g). The expensive, weak responder Q-BC (peak yield 2.56 g·L^−1^) reached comparative performance with a value range of 13.43–17.16 g/€.

W-BC, BY-BC and SG-BC constituted the third, moderately performing cereal group (peak yields 1.70–2.21 g·L^−1^), with cost efficiency values of 6.01–16.46 g/€. Just like in the case of the third group under the CTN substitution regime, these media formulations also improved productivity compared to the control based on their acceptable yields and low bulk prices (0.06–0.07 €/g).

The fourth, weakly performing group (peak yields 0.54–0.66 g·L^−1^) was made up of RY-BC, M-BC and C-BC, all cereals with weak substitution responses and low cost efficiency values of 1.55–5.49 g/€. This group underperformed the control at all substitution levels under the CNSM regime.

For the first two groups presented above, BC unit production cost decreased substantially relative to the corresponding CNSM HS control, with the maximum reduction observed for TR-BC at 75% substitution (0.26 to 0.08 €/g; 68.40%; Table S4B). The third group reached its maximum reduction for BY-BC at 50% substitution (0.25 to 0.07 €/g; 71.52%).

There was significant variation in the cost efficiency of certain top-performing flours depending on the substitution regime. While the comparatively low-nitrogen W-BC, C-BC and M-BC flours had very good results under CTN, they were among the worst performers under CNSM. The same inversion was observed for the similarly low-nitrogen O-BC and R-BC flours, which performed well under CNSM but poorly under CTN. This variability is probably related to rheological and oxygen-transfer limitations associated with higher solids concentrations in static BC cultures [28,31].

TR-BC and SP-BC, two high-nitrogen but low-priced flours, maintained good performance under both regimes. It is noteworthy that the same is true for high-priced high-nitrogen flours like S-BC, Q-BC and T-BC. The extent to which these components boosted production outweighed the media cost increase caused by their high prices.

The techno-economic analysis highlights a critical distinction between apparent cost efficiency (g/€) and actual unit cost reduction (%). While several formulations, such as O-BC and T-BC under CTN or S-BC and B-BC under CNSM, exhibited moderate or high-cost efficiency, these did not necessarily translate into reduced unit BC cost compared to the control. Accordingly, economic benefit was attributed only to formulations demonstrating clear unit cost reduction.

Overall, TEA demonstrates that the viability of flour-based media for bacterial cellulose production is determined by the combined influence of feedstock cost and biological compatibility with *Komagataeibacter*. In this context, alongside, some low-cost cereals exhibiting moderate but consistent BC yields (e.g., BY-BC, SG-BC and TR-BC) became economically more favorable than high-yield cereals such as SP-BC after normalization. This trend reflects a common outcome in techno-economic evaluations, where feedstock price can exert a greater influence on overall process economics than product yield [27]. Some flours associated with high BC productivity, such as O-BC and T-BC, did not consistently indicate economic advantages, particularly under CNSM conditions, likely due to increased viscosity and mass-transfer limitations, process constraints which can suppress yield. These observations align with prior techno-economic analysis in BC and related fermentation systems, which reported that laboratory-scale yield was unable to compensate for unfavorable substrate costs or process limitations that reduce the efficiency of nutrient utilization [23,24].

The favorable performance of low-cost flour is consistent with previous studies on cereal-derived residues, which reported substantial reductions during medium substitution and thus emphasized the role of medium composition and solids concentration in overall production [21,24]. These results highlight the potential for further optimization through targeted strategies such as mild pretreatment, improved aeration, or strategic blending of low-cost and high-yield flours to enhance both BC productivity and cost efficiency in scalable production systems. In a previous article, the structural characteristics of flour-derived BC, including fibrillar morphology and crystallinity, were also examined in detail [20].

The present techno-economic assessment is restricted to medium formulation cost and does not include operating expenditures such as energy demand, labor, or downstream purification. A comprehensive industrial evaluation would require research beyond the scope of this work. Nevertheless, because refined nitrogen supplements constitute a major cost driver in HS-based media, the evaluated media formulations represent a potential path for improving the economics of BC production.

Techno-economic evaluation in BC research is most performed retrospectively, following process optimization [24]. In contrast, the present study integrates cost normalization prospectively at the experimental design stage, enabling for the evaluation of substitution strategies prior to process optimization. While previous reports have shown how low-cost agro-products or residues can reduce medium expenses, quantitative normalization to unit BC production cost (€/g) was rarely presented alongside biological performance. By coupling graded nitrogen substitution with cost-normalized yield assessment, this work provides a potential framework for early-stage economic screening of fermentation media formulations.

## 4. Conclusions

This study demonstrates that agro-derived flours, applied under CTN and CNSM substitution regimes, can partially replace HS as a medium for BC production while maintaining and, in several cases, improving BC productivity during the fermentation process of *K. xylinus*. Across the fourteen flours evaluated, BC yields obtained in flour-based media were generally comparable to those of the corresponding controls, and in some cases they exceeded them. Under CTN, the highest yields were obtained for W-BC, C-BC, M-BC and SP-BC (6.68–8.97 g·L^−1^), whereas under CNSM, the highest yields were recorded for O-BC and T-BC (4.24–5.14 g·L^−1^). Yield changes relative to the corresponding controls were classified as “strong”, “moderate” and “weak”. Under CTN, SP-BC, Q-BC, TR-BC, SG-BC and BY-BC all achieved yield increases of at least 100%, with the maximum increase observed for TR-BC (190%). Under CNSM, the highest increase was recorded for BY-BC (149%).

Using a violin plot and Spearman’s correlation heatmap, analysis revealed flour-specific optima and highlighted a general advantage of intermediate substitution levels over complete replacement, particularly under the CNSM regime, where the fixed media weight concentration increasingly constrained BC yields at higher substitution levels. It was also shown that some flours such as TR-BC and SP-BC were still able to maintain good performance even at high substitution levels.

Techno-economic assessment demonstrated that cost efficiency (g/€) and unit cost reduction (%) do not necessarily correlate, as several low-cost cereals (e.g., BY-BC, SG-BC and TR-BC) with moderate BC yields reduced BC unit production cost more effectively than some high-yield and high-cost-efficiency cereals (e.g., SP-BC, O-BC and T-BC). Under CTN, the maximum unit cost reduction was observed for TR-BC at 75% substitution, where BC unit production cost decreased from 0.27 to 0.08 €/g relative to the corresponding CTN HS control, representing a 70.37% reduction. Under CNSM, the maximum reduction was observed for BY-BC at 50% substitution, where BC unit production cost decreased from 0.25 to 0.07 €/g relative to the corresponding CNSM HS control, corresponding to a 72.00% reduction.

However, conclusions regarding BC quality preservation apply only to the selected high-yielding formulations previously characterized against the corresponding HS controls and should not be generalized to all flour-containing media.

These findings indicate that graded nitrogen substitution at moderate substitution levels (25–50%), particularly under CTN conditions, represents a robust and economically effective strategy for reducing reliance on refined nitrogen supplements while supporting scalable and sustainable BC production. Furthermore, the flour selection framework used for this study can present a viable model for the evaluation of substitution strategies prior to process optimization for BC production.

## Figures and Tables

**Table 1:** Overview of flour quantities used under the constant total nitrogen (CTN, 0.6 g·L^−1^) and constant nitrogen-source mass (CNSM, 5 g·L^−1^) regimes at 25%, 50%, and 75% substitution levels.

A. Constant total nitrogen (CTN)—0.6 g·L^−1^ fixed nitrogen content				
Media Component (g·L^−1^)	0% (Control)	25%	50%	75%
Sucrose	80	80	80	80
Yeast Extract (value dependent on flour type)	2.5	2.0–2.43	1.0–2.30	0.5–1.95
Tryptone (value dependent on flour type)	2.5	2.00–2.43	1.0–2.30	0.5–1.95
S-Flour	0	1.74	5.22	6.96
C-Flour	0	1.61	4.48	11.11
R-Flour	0	1.58	4.60	11.50
W-Flour	0	1.58	4.28	9.93
O-Flour	0	1.62	4.32	10.19
SP-Flour	0	1.55	4.20	9.65
Q-Flour	0	1.60	4.26	9.80
B-Flour	0	1.58	4.30	10.26
T-Flour	0	1.60	4.25	9.00
M-Flour	0	1.58	4.30	10.26
TR-Flour	0	1.60	4.20	9.60
RY-Flour	0	1.62	4.40	10.80
BY-Flour	0	1.62	4.38	10.73
SG-Flour	0	1.62	4.44	10.82
B. Constant nitrogen-source mass (CNSM)—5 g·L^−1^ fixed media weight				
Media Component (g·L^−1^)	0% (Control)	25%	50%	75%
Sucrose	80	80	80	80
Yeast Extract	2.5	1.875	1.25	0.625
Tryptone	2.5	1.875	1.25	0.625
All Flour Types	0	1.25	2.50	3.75

**Table 2:** Comparative general response patterns of flour substrates under CTN and CNSM regimes. Data represents triplicate measurements at 25%, 50%, and 75% substitution *. Abbreviations: S (soy), C (corn), W (wheat), R (rice), O (oat), B (bulgur), SP (spelt), Q (quinoa), T (teff), M (millet), TR (triticale), RY (rye), SG (sorghum), BY (barley).

Flour (Code)	CTN (0.6 g·L^−1^)	CNSM (5 g·L^−1^)	Notes
S-BC	Moderate	Weak	Small CTN increments (0.32 g·L^−1^ highest), no increment under CNSM
C-BC	Weak	Weak	Small CTN increments (3.70 g·L^−1^ highest), no increment under CNSM; low nitrogen and low soluble nutrient fraction
W-BC	Moderate	Weak	Moderate CTN increments (4.03 g·L^−1^ highest), low performance under CNSM
R-BC	Weak	Moderate	No increment under CTN, medium CNSM increment at 25% and 50% substitution levels (0.96 g·L^−1^ highest)
O-BC	Weak	Weak	Worse than control in all but one case (highest increment 1.06 g·L^−1^)
B-BC	Weak	Weak	Worse than control in all but one case (highest increment 0.26 g·L^−1^)
SP-BC	Strong	Weak	High increments under CTN (3.37 g·L^−1^ highest), maintains performance under CNSM but yield does not increase
Q-BC	Strong	Weak	High increments under CTN (2.67 g·L^−1^ highest), low performance under CNSM
T-BC	Weak	Moderate	No increase under CTN, medium increments under CNSM (1.61 g·L^−1^ highest); high protein and micronutrient content nevertheless
M-BC	Moderate	Weak	Medium increment under CTN (3.47 g·L^−1^); poor performer under CNSM and solids stress.
TR-BC	Strong	Moderate	One of the strongest performers under CTN and CNSM (1.60·L^−1^ highest)
RY-BC	Weak	Weak	No increment under both regimes
SG-BC	Strong	Moderate	Strong performer under both CTN and CNSM (1.34·L^−1^ highest)
BY-BC	Strong	Strong	Strong performer under both regimes (1.42·L^−1^ highest)

* Classification based on increment values across 25%, 50%, and 75% substitution.

## Data Availability

The data supporting the findings of this study are available within the article and the Supplementary Materials.

## Supplementary Materials

The following supporting information can be downloaded at: https://www.mdpi.com/article/10.3390/polym18060721/s1, Table S1: Bacterial cellulose yields in flour-substituted media under constant total nitrogen and constant nitrogen source mass conditions; Table S2: Initial and final pH values of flour-substituted media under constant total nitrogen and constant nitrogen source mass conditions; Table S3: Techno-economic input parameters, total medium cost, and cost efficiency of bacterial cellulose production in flour-substituted media under constant total nitrogen and constant nitrogen source mass conditions; Table S4: Unit production cost and relative cost reduction of bacterial cellulose produced in flour-substituted media compared with the Hestrin–Schramm control; Table S5: Nitrogen and protein contents of the tested flour variants determined from literature values and by the Kjeldahl method.

## Abbreviations

The following abbreviations are used in this manuscript:

BC	Bacterial Cellulose
C/N	Carbon-to-Nitrogen (ratio)
CNSM	Constant Nitrogen-Source Mass
CTN	Constant Total Nitrogen
HS	Hestrin–Schramm
SZMC	Szeged Microbiology Collection
TEA	Techno-Economic Analysis
