# Supplementary material for: Techno-Economic and Statistical Assessment of Agricultural Flours for Bacterial Cellulose Production by Komagataeibacter xylinus
Source: Polymers (Basel). 2026 Mar 16;18(6):721. doi: 10.3390/polym18060721 (PMC13030736; doi:10.3390/polym18060721)
Supplement: Supplementary file 1 [file polymers-18-00721-s001.zip › polymers-4170308-supplementary.pdf]

SUPPLEMENTARY INFORMATION

Techno-Economic and Statistical Assessment of Agricultural Flours for Bacterial Cellulose Production by *Komagataeibacter xylinus*

Dheanda Absharina, Csilla Veres, Sándor Kocsubé, and Csaba Vágvölgyi*

Supplementary Table S1. BC yields for flour-substituted media under Constant Total Nitrogen (CTN) (0.6 $g{\cdot}L^{-1}$ fixed nitrogen content) and Constant Nitrogen Source Mass (CNSM) (5 $g{\cdot}L^{-1}$ fixed flour mass).
Data represents 25%, 50%, and 75% substitution. Abbreviations: S (soy), C (corn), W (wheat), R (rice), O (oat), B (bulgur), SP (spelt), Q (quinoa), T (teff), M (millet), TR (triticale), RY (rye), SG (sorghum), BY (barley). Values are reported as BC yield ($g.L^{-1}$).

Table S1A. BC yield under Constant Total Nitrogen (CTN: 0.6 $g{\cdot}L^{-1}$ fixed nitrogen).

Flour (Code)	25% BC yield ($g{\cdot}L^{-1}$)	50% BC yield ($g{\cdot}L^{-1}$)	75% BC yield ($g{\cdot}L^{-1}$)
S-BC	2.49	2.66	2.95
C-BC	5.19	7.92	4.41
W-BC	6.14	8.97	5.75
R-BC	1.49	1.36	1.71
O-BC	4.63	2.15	2.16
B-BC	1.85	4.30	3.93
SP-BC	5.53	6.68	5.37
Q-BC	2.48	2.97	5.00
T-BC	3.20	2.60	4.25
M-BC	3.54	7.51	5.75
TR-BC	1.87	2.44	2.16
RY-BC	1.94	1.47	2.39
SG-BC	2.33	2.40	1.00
BY-BC	1.60	2.31	1.74

Table S1B. BC yield under Constant Nitrogen Source Mass (CNSM: 5 $g{\cdot}L^{-1}$ fixed flour mass).

Flour (Code)	25% BC yield ($g{\cdot}L^{-1}$)	50% BC yield ($g{\cdot}L^{-1}$)	75% BC yield ($g{\cdot}L^{-1}$)
S-BC	2.37	2.11	2.46
C-BC	0.56	0.55	0.64
W-BC	1.13	1.15	1.92
R-BC	2.92	3.18	1.67
O-BC	3.73	5.14	2.64
B-BC	3.24	2.46	1.91
SP-BC	3.27	3.31	3.48
Q-BC	2.56	2.25	2.16
T-BC	4.24	3.59	2.84
M-BC	0.66	0.45	0.32
TR-BC	1.12	1.11	1.41
RY-BC	0.54	0.47	0.18
SG-BC	1.70	1.43	0.70
BY-BC	2.21	2.13	1.24

Supplementary Table S2.

Fermentation pH values for flour-substituted media under Constant Total Nitrogen (CTN) (0.6 g·L^{-1} fixed nitrogen content) and Constant Nitrogen Source Mass (CNSM) (5 g·L^{-1} fixed flour mass). Data represents 25%, 50%, and 75% substitution (100% where applicable). Values are reported as pH at inoculation (pH_0) and after 7 days of static cultivation (pH_7).

Supplementary Table S2A. Fermentation pH under Constant Total Nitrogen (CTN: 0.6 g·L^{-1} fixed nitrogen).

Flour	Substitution (%)	Initial pH (pH_0)	Final pH (pH_7)
S-BC	25	6	4.3
S-BC	50	6	4.3
S-BC	75	6	4
C-BC	25	6	5.2
C-BC	50	6	4.9
C-BC	75	6	4.7
R-BC	25	5	4
R-BC	50	5	4
R-BC	75	5	4
W-BC	25	6	5.2
W-BC	50	6	4.9
W-BC	75	5.2	4.9
O-BC	25	6	4
O-BC	50	6	4
O-BC	75	6	4
SP-BC	25	6	3.8
SP-BC	50	6	3.8
SP-BC	75	6	3.8
Q-BC	25	5.5	4
Q-BC	50	5.5	4
Q-BC	75	5.5	4
B-BC	25	5.8	4.3
B-BC	50	5.8	4.3
B-BC	75	5.8	4.3
T-BC	25	5.5	4.3
T-BC	50	5.5	4.3
T-BC	75	5.5	4
M-BC	25	5.8	4.3
M-BC	50	5.2	4.3
M-BC	75	5.2	4.3
TR-BC	25	5.8	3.8
TR-BC	50	5.5	3.8
TR-BC	75	5.5	3.8
RY-BC	25	6.5	4.3
RY-BC	50	6.5	4.3
RY-BC	75	6	4
BY-BC	25	5.8	4.3
BY-BC	50	5.2	4.3
BY-BC	75	5.2	4.3
SG-BC	25	6.5	4.3
SG-BC	50	6.5	4.3
SG-BC	75	6	4.3

Supplementary Table S2B. Fermentation pH under Constant Nitrogen Source Mass (CNSM: 5 g·L^{-1} fixed flour mass).

Flour	Substitution (%)	Initial pH (pH_0)	Final pH (pH_7)
S-BC	25	6.5	5
S-BC	50	6.5	5
S-BC	75	6	5
C-BC	25	6	4
C-BC	50	6	4
C-BC	75	6	4
R-BC	25	6	4
R-BC	50	5.8	4
R-BC	75	5.5	4
W-BC	25	5.2	4
W-BC	50	6	4.3
W-BC	75	6	4.3
O-BC	25	5.2	4.3
O-BC	50	6.1	4
O-BC	75	6.1	4
SP-BC	25	6.1	4
SP-BC	50	6.1	3.8
SP-BC	75	6.1	3.8
Q-BC	25	6.1	3.8
Q-BC	50	5.5	4
Q-BC	75	5.5	4
B-BC	25	5.5	4
B-BC	50	5.8	4.3
B-BC	75	5.2	4.3
T-BC	25	5.2	4.3
T-BC	50	5.5	4.3
T-BC	75	5.5	4.3
M-BC	25	5.5	4
M-BC	50	5.5	3.8
M-BC	75	5.2	3.8
TR-BC	25	5.2	3.8
TR-BC	50	5.2	3.8
TR-BC	75	5.2	3.8
RY-BC	25	5.2	3.8
RY-BC	50	5.8	3.8
RY-BC	75	5.2	3.8
BY-BC	25	5.2	3.8
BY-BC	50	5.8	3.8
BY-BC	75	5.2	3.8
SG-BC	25	5.2	3.8
SG-BC	50	6.5	4.3
SG-BC	75	6.5	4.3

Supplementary Table S3. Techno-economic input and output parameters for flour-substituted media under CTN and CNSM regimes.

Table S3A. BC yield and cost-efficiency under Constant Total Nitrogen (CTN: 0.6 g·L^{-1} fixed nitrogen).

Flour (Code)	Subs. level	BC yield (g·L^{-1})	Cereal cost (€)	HS media cost (€)	Sucrose cost (€)	Total cost (€)	Cost efficiency (g/€)	Control pellicle weight (g)	Control total cost (€)	Control cost efficiency (g/€)
R-BC	25%	1.49	0.0008	0.1385	0.0802	0.2195	6.79	2.01	0.22	9.01
R-BC	50%	1.36	0.0023	0.1313	0.0802	0.2139	6.36	2.01	0.22	9.01
R-BC	75%	1.71	0.0058	0.1114	0.0802	0.1973	8.67	2.01	0.22	9.01
M-BC	25%	3.54	0.0005	0.1353	0.0802	0.2160	16.39	4.04	0.22	18.12
M-BC	50%	7.51	0.0013	0.1228	0.0802	0.2043	36.75	4.04	0.22	18.12
M-BC	75%	5.75	0.0032	0.0977	0.0802	0.1811	31.76	4.04	0.22	18.12
O-BC	25%	4.63	0.0003	0.1353	0.0802	0.2158	21.45	4.08	0.22	18.30
O-BC	50%	2.15	0.0007	0.1233	0.0802	0.2043	10.52	4.08	0.22	18.30
O-BC	75%	2.16	0.0017	0.0968	0.0802	0.1787	12.08	4.08	0.22	18.30
RY-BC	25%	1.94	0.0002	0.1368	0.0802	0.2172	8.93	3.30	0.22	14.80
RY-BC	50%	1.47	0.0006	0.1268	0.0802	0.2076	7.08	3.30	0.22	14.80
RY-BC	75%	2.39	0.0016	0.1051	0.0802	0.1869	12.79	3.30	0.22	14.80
SG-BC	25%	2.33	0.0003	0.1368	0.0802	0.2172	10.73	1.06	0.22	4.75
SG-BC	50%	2.40	0.0007	0.1268	0.0802	0.2077	11.55	1.06	0.22	4.75
SG-BC	75%	1.00	0.0018	0.1051	0.0802	0.1870	5.35	1.06	0.22	4.75
BY-BC	25%	1.60	0.0002	0.1368	0.0802	0.2172	7.37	0.89	0.22	3.99
BY-BC	50%	2.31	0.0008	0.1262	0.0802	0.2071	11.16	0.89	0.22	3.99
BY-BC	75%	1.74	0.0019	0.1056	0.0802	0.1875	9.28	0.89	0.22	3.99
SP-BC	25%	5.53	0.0003	0.1353	0.0802	0.2159	25.62	3.31	0.22	14.84
SP-BC	50%	6.68	0.0008	0.1199	0.0802	0.2009	33.24	3.31	0.22	14.84
SP-BC	75%	5.37	0.0019	0.0902	0.0802	0.1723	31.16	3.31	0.22	14.84
B-BC	25%	1.85	0.0005	0.1353	0.0802	0.2160	8.56	4.04	0.22	18.12
B-BC	50%	4.30	0.0012	0.1228	0.0802	0.2042	21.06	4.04	0.22	18.12
B-BC	75%	3.93	0.0029	0.0977	0.0802	0.1808	21.74	4.04	0.22	18.12
TR-BC	25%	1.87	0.0003	0.1342	0.0802	0.2148	8.71	0.84	0.22	3.77
TR-BC	50%	2.44	0.0009	0.1199	0.0802	0.2010	12.14	0.84	0.22	3.77
TR-BC	75%	2.16	0.0021	0.0925	0.0802	0.1748	12.36	0.84	0.22	3.77
T-BC	25%	3.20	0.0024	0.1342	0.0802	0.2168	14.76	5.26	0.22	23.59
T-BC	50%	2.60	0.0064	0.1213	0.0802	0.2079	12.50	5.26	0.22	23.59
T-BC	75%	4.25	0.0135	0.0971	0.0802	0.1908	22.28	5.26	0.22	23.59
Q-BC	25%	2.48	0.0043	0.1342	0.0802	0.2187	11.34	2.33	0.22	10.45
Q-BC	50%	2.97	0.0114	0.1216	0.0802	0.2132	13.93	2.33	0.22	10.45
Q-BC	75%	5.00	0.0261	0.0942	0.0802	0.2006	24.93	2.33	0.22	10.45
S-BC	25%	2.49	0.0008	0.1142	0.0802	0.1952	12.76	2.63	0.22	11.80
S-BC	50%	2.66	0.0024	0.0571	0.0802	0.1397	19.04	2.63	0.22	11.80
S-BC	75%	2.95	0.0032	0.0286	0.0802	0.1119	26.35	2.63	0.22	11.80
C-BC	25%	5.19	0.0003	0.1373	0.0802	0.2178	23.83	4.22	0.22	18.93
C-BC	50%	7.92	0.0008	0.1279	0.0802	0.2089	37.91	4.22	0.22	18.93
C-BC	75%	4.41	0.0020	0.1056	0.0802	0.1878	23.48	4.22	0.22	18.93
W-BC	25%	6.14	0.0003	0.1353	0.0802	0.2158	28.45	4.94	0.22	22.16
W-BC	50%	8.97	0.0008	0.1222	0.0802	0.2032	44.13	4.94	0.22	22.16
W-BC	75%	5.75	0.0019	0.0954	0.0802	0.1775	32.39	4.94	0.22	22.16

Web-based sources for media components and agro-derived substrate costs are cited in the main manuscript References.

Table S3B. BC yield and cost-efficiency under Constant Nitrogen Source Mass (CNSM: 5 g·L^{-1} flour).

Flour (Code)	Subs. level	BC yield (g·L^{-1})	Cereal cost (€)	HS media cost (€)	Sucrose cost (€)	Total cost (€)	Cost efficiency (g/€)	Control pellicle weight (g)	Control total cost (€)	Control cost efficiency (g/€)
R-BC	25%	2.92	0.0006	0.1071	0.0802	0.1879	15.54	2.22	0.22	9.96
R-BC	50%	3.18	0.0013	0.0714	0.0802	0.1528	20.81	2.22	0.22	9.96
R-BC	75%	1.67	0.0019	0.0357	0.0802	0.1178	14.18	2.22	0.22	9.96
M-BC	25%	0.66	0.0004	0.1071	0.0802	0.1877	3.52	0.84	0.22	3.77
M-BC	50%	0.45	0.0008	0.0714	0.0802	0.1524	2.95	0.84	0.22	3.77
M-BC	75%	0.32	0.0012	0.0357	0.0802	0.1171	2.73	0.84	0.22	3.77
O-BC	25%	3.73	0.0002	0.1071	0.0802	0.1875	19.89	4.08	0.22	18.30
O-BC	50%	5.14	0.0004	0.0714	0.0802	0.1520	33.81	4.08	0.22	18.30
O-BC	75%	2.64	0.0006	0.0357	0.0802	0.1165	22.65	4.08	0.22	18.30
RY-BC	25%	0.54	0.0002	0.1071	0.0802	0.1875	2.88	1.27	0.22	5.70
RY-BC	50%	0.47	0.0004	0.0714	0.0802	0.1520	3.09	1.27	0.22	5.70
RY-BC	75%	0.18	0.0006	0.0357	0.0802	0.1165	1.55	1.27	0.22	5.70
SG-BC	25%	1.70	0.0002	0.1071	0.0802	0.1875	9.07	1.06	0.22	4.75
SG-BC	50%	1.43	0.0004	0.0714	0.0802	0.1520	9.41	1.06	0.22	4.75
SG-BC	75%	0.70	0.0006	0.0357	0.0802	0.1165	6.01	1.06	0.22	4.75
BY-BC	25%	2.21	0.0002	0.1071	0.0802	0.1875	11.79	0.89	0.22	3.99
BY-BC	50%	2.13	0.0004	0.0714	0.0802	0.1520	14.02	0.89	0.22	3.99
BY-BC	75%	1.24	0.0006	0.0357	0.0802	0.1165	10.65	0.89	0.22	3.99
SP-BC	25%	3.27	0.0002	0.1071	0.0802	0.1875	17.44	3.31	0.22	14.84
SP-BC	50%	3.31	0.0005	0.0714	0.0802	0.1521	21.77	3.31	0.22	14.84
SP-BC	75%	3.48	0.0007	0.0357	0.0802	0.1166	29.34	3.31	0.22	14.84
B-BC	25%	3.24	0.0004	0.1071	0.0802	0.1876	17.27	4.04	0.22	18.12
B-BC	50%	2.46	0.0007	0.0714	0.0802	0.1523	16.15	4.04	0.22	18.12
B-BC	75%	1.91	0.0011	0.0357	0.0802	0.1170	16.32	4.04	0.22	18.12
TR-BC	25%	1.12	0.0003	0.1071	0.0802	0.1876	5.97	0.84	0.22	3.77
TR-BC	50%	1.11	0.0005	0.0714	0.0802	0.1521	7.30	0.84	0.22	3.77
TR-BC	75%	1.41	0.0008	0.0357	0.0802	0.1167	12.08	0.84	0.22	3.77
T-BC	25%	4.24	0.0019	0.1071	0.0802	0.1892	22.42	2.63	0.22	11.80
T-BC	50%	3.59	0.0038	0.0714	0.0802	0.1553	23.11	2.63	0.22	11.80
T-BC	75%	2.84	0.0056	0.0357	0.0802	0.1215	23.37	2.63	0.22	11.80
Q-BC	25%	2.56	0.0033	0.1071	0.0802	0.1906	13.43	2.71	0.22	12.15
Q-BC	50%	2.25	0.0067	0.0714	0.0802	0.1583	14.22	2.71	0.22	12.15
Q-BC	75%	2.16	0.0100	0.0357	0.0802	0.1259	17.16	2.71	0.22	12.15
S-BC	25%	2.37	0.0006	0.1071	0.0802	0.1879	12.62	2.67	0.22	11.97
S-BC	50%	2.11	0.0011	0.0714	0.0802	0.1527	13.81	2.67	0.22	11.97
S-BC	75%	2.46	0.0017	0.0357	0.0802	0.1176	20.92	2.67	0.22	11.97
C-BC	25%	0.56	0.0002	0.1071	0.0802	0.1875	2.99	1.71	0.22	7.67
C-BC	50%	0.55	0.0004	0.0714	0.0802	0.1520	3.62	1.71	0.22	7.67
C-BC	75%	0.64	0.0007	0.0357	0.0802	0.1166	5.49	1.71	0.22	7.67
W-BC	25%	1.13	0.0002	0.1071	0.0802	0.1875	6.03	1.88	0.22	8.43
W-BC	50%	1.15	0.0005	0.0714	0.0802	0.1521	7.56	1.88	0.22	8.43
W-BC	75%	1.92	0.0007	0.0357	0.0802	0.1166	16.46	1.88	0.22	8.43

Web-based sources for media components and agro-derived substrate costs are cited in the main manuscript References.

Supplementary Table S4. Cost reduction (%) calculated relative to the corresponding Hestrin–Schramm control based on unit production cost (€/g).

Table S4A. Unit cost of BC production under Constant Total Nitrogen (CTN: 0.6 g·L^{-1} fixed nitrogen).

Flour (Code)	Subs. level	Control cost efficiency (g/€)	Control BC unit production cost (€/g)	Cost efficienc y (g/€)	BC unit production cost (€/g)	Unit cost reduction (%)
R-BC	25%	9.01	0.11	6.79	0.15	-
R-BC	50%	9.01	0.11	6.36	0.16	-
R-BC	75%	9.01	0.11	8.67	0.12	-
M-BC	25%	18.12	0.06	16.39	0.06	-
M-BC	50%	18.12	0.06	36.75	0.03	50.00%
M-BC	75%	18.12	0.06	31.76	0.03	50.00%
O-BC	25%	18.30	0.05	21.45	0.05	-
O-BC	50%	18.30	0.05	10.52	0.10	-
O-BC	75%	18.30	0.05	12.08	0.08	-
RY-BC	25%	14.80	0.07	8.93	0.11	-
RY-BC	50%	14.80	0.07	7.08	0.14	-
RY-BC	75%	14.80	0.07	12.79	0.08	-
SG-BC	25%	4.75	0.21	10.73	0.09	57.14%
SG-BC	50%	4.75	0.21	11.55	0.09	57.14%
SG-BC	75%	4.75	0.21	5.35	0.19	9.52%
BY-BC	25%	3.99	0.25	7.37	0.14	44.00%
BY-BC	50%	3.99	0.25	11.16	0.09	64.00%
BY-BC	75%	3.99	0.25	9.28	0.11	56.00%
SP-BC	25%	14.84	0.07	25.62	0.04	42.86%
SP-BC	50%	14.84	0.07	33.24	0.03	57.14%
SP-BC	75%	14.84	0.07	31.16	0.03	57.14%
B-BC	25%	18.12	0.06	8.56	0.12	-
B-BC	50%	18.12	0.06	21.06	0.05	16.67%
B-BC	75%	18.12	0.06	21.74	0.05	16.67%
TR-BC	25%	3.77	0.27	8.71	0.11	59.26%
TR-BC	50%	3.77	0.27	12.14	0.08	70.37%
TR-BC	75%	3.77	0.27	12.36	0.08	70.37%
T-BC	25%	23.59	0.04	14.76	0.07	-
T-BC	50%	23.59	0.04	12.50	0.08	-
T-BC	75%	23.59	0.04	22.28	0.04	-
Q-BC	25%	10.45	0.10	11.34	0.09	10.00%
Q-BC	50%	10.45	0.10	13.93	0.07	30.00%
Q-BC	75%	10.45	0.10	24.93	0.04	60.00%
S-BC	25%	11.80	0.08	12.76	0.08	-
S-BC	50%	11.80	0.08	19.04	0.05	37.50%
S-BC	75%	11.80	0.08	26.35	0.04	50.00%
C-BC	25%	18.93	0.05	23.83	0.04	20.00%
C-BC	50%	18.93	0.05	37.91	0.03	40.00%
C-BC	75%	18.93	0.05	23.48	0.04	20.00%
W-BC	25%	22.16	0.05	28.45	0.04	20.00%
W-BC	50%	22.16	0.05	44.13	0.02	60.00%
W-BC	75%	22.16	0.05	32.39	0.03	40.00%

("–" indicates no cost reduction relative to the Hestrin–Schramm control.)*

Table S4B. Unit cost of BC production under Constant Nitrogen Source Mass (CNSM: 5 g·L^{-1} flour).

Flour (Code)	Subs. level	Control cost efficiency (g/€)	Control BC unit production cost (€/g)	Cost efficienc y (g/€)	BC unit production cost (€/g)	Unit cost reduction (%)
R-BC	25%	9.96	0.10	15.54	0.06	40.00%
R-BC	50%	9.96	0.10	20.81	0.05	50.00%
R-BC	75%	9.96	0.10	14.18	0.07	30.00%
M-BC	25%	3.77	0.27	3.52	0.28	-
M-BC	50%	3.77	0.27	2.95	0.34	-
M-BC	75%	3.77	0.27	2.73	0.37	-
O-BC	25%	18.30	0.05	19.89	0.05	-
O-BC	50%	18.30	0.05	33.81	0.03	40.00%
O-BC	75%	18.30	0.05	22.65	0.04	20.00%
RY-BC	25%	5.70	0.18	2.88	0.35	-
RY-BC	50%	5.70	0.18	3.09	0.32	-
RY-BC	75%	5.70	0.18	1.55	0.65	-
SG-BC	25%	4.75	0.21	9.07	0.11	47.62%
SG-BC	50%	4.75	0.21	9.41	0.11	47.62%
SG-BC	75%	4.75	0.21	6.01	0.17	19.05%
BY-BC	25%	3.99	0.25	11.79	0.08	68.00%
BY-BC	50%	3.99	0.25	14.02	0.07	72.00%
BY-BC	75%	3.99	0.25	10.65	0.09	64.00%
SP-BC	25%	14.84	0.07	17.44	0.06	14.29%
SP-BC	50%	14.84	0.07	21.77	0.05	28.57%
SP-BC	75%	14.84	0.07	29.34	0.03	57.14%
B-BC	25%	18.12	0.06	17.27	0.06	-
B-BC	50%	18.12	0.06	16.15	0.06	-
B-BC	75%	18.12	0.06	16.32	0.06	-
TR-BC	25%	3.77	0.27	5.97	0.17	37.04%
TR-BC	50%	3.77	0.27	7.30	0.14	48.15%
TR-BC	75%	3.77	0.27	12.08	0.08	70.37%
T-BC	25%	11.80	0.08	22.42	0.04	50.00%
T-BC	50%	11.80	0.08	23.11	0.04	50.00%
T-BC	75%	11.80	0.08	23.37	0.04	50.00%
Q-BC	25%	12.15	0.08	13.43	0.07	12.50%
Q-BC	50%	12.15	0.08	14.22	0.07	12.50%
Q-BC	75%	12.15	0.08	17.16	0.06	25.00%
S-BC	25%	11.97	0.08	12.62	0.08	-
S-BC	50%	11.97	0.08	13.81	0.07	12.50%
S-BC	75%	11.97	0.08	20.92	0.05	37.50%
C-BC	25%	7.67	0.13	2.99	0.33	-
C-BC	50%	7.67	0.13	3.62	0.28	-
C-BC	75%	7.67	0.13	5.49	0.18	-
W-BC	25%	8.43	0.12	6.03	0.17	-
W-BC	50%	8.43	0.12	7.56	0.13	-
W-BC	75%	8.43	0.12	16.46	0.06	50.00%

("–" indicates no cost reduction relative to the Hestrin–Schramm control.)*

Supplementary Table S5. Total nitrogen content was determined using literature values. Nitrogen and crude protein content were also obtained experimentally using the Kjeldahl method with flour-specific nitrogen-to-protein conversion factors, for the purpose of comparison. Adapted from Ref. [20].

No	Flour Variant	Nitrogen % (from literature)	Nitrogen % (Kjeldahl)*	Protein % (from literature)	Protein % (Kjeldahl)*
1	Soy	6.90%	7.22%	38.6%; 50.00%	44.52%
2	Corn	1.40%	1.08%	7.56%	7.39%
3	Wheat	2.01%	1.75%	9-14%	10.79%
4	Rice	1.11%	1.17%	6.91 %	7.83%
5	Oat	1.90%	1.28%	12.50%;13.50%	8.44%
6	Bulgur	1.88%	1.81%	12.32 %	11.82%
7	Millet	1.85%	1.94%	10.75%	12.25%
8	Spelt	2.32	2.62%	14.50%; 16.60-20.00%	16.67%
9	Quinoa	2.04%	2.09%	13.50%	13.15%
10	Triticale	2.25%	1.72%	9.80-13.90%	11.11%
11	Teff	2.13%	1.20%	9.07%; 10.21-13.30%	8.47%
12	Rye	1.44%	1.58%	6.50-9.00%; 8.40%	10.01%
13	Sorghum	1.42%	1.24%	7.70%	7.78%
14	Barley	1.5%	1.44%	8.72%	9.46%
